# Colour Order

**DOI:** 10.1177/2041669519872516

**Published:** 2019-09-04

**Authors:** Jan Koenderink, Andrea van Doorn, Karl Gegenfurtner

**Affiliations:** Justus Liebig University Giessen, Germany; University of Leuven (KU Leuven), Belgium; Utrecht University, the Netherlands; Justus Liebig University Giessen, Germany; Utrecht University, the Netherlands; Justus Liebig University Giessen, Germany

**Keywords:** colour sorting, hundred hue test, hue circle, hue resolution

## Abstract

Scrambled hue circles with a resolution ranging from 6 steps to 60 steps were presented on a varicoloured background. The hue steps were presented as mutually non-contiguous “chips,” small circular disks, placed uniformly on a large circle. The task was to sort the chips with respect to their hue. Participants generally manage to sort a 24-step hue circle faultlessly but commit many ordering reversals (also of several steps, up to five) on sorting a 60-step hue circle. The pattern of local reversals of chips depends on the hue region. The findings are relevant for the design of user interfaces for various types of applications, such as colour pickers or graphical design, that rely on rgb screen colours as the available palette.

## Introduction

In this article, we limit “colours” to “object colours” and—especially—“(rgb) screen colours.” For object colours, the radiant spectra scattered to the eye are—on a per wavelength basis—dominated by the radiant spectrum scattered to the eye by a Lambertian surface of unit albedo and a standard illuminant. As illuminant one picks “natural daylight” (e.g., cie illuminant d65). For the rgb screen colours, the rgb coordinates are dominated by those of the screen “white.”^1^

The object colours are contained in a convex body, the “object colour solid” in (say cie XYZ or any basis) colour space, whereas the screen colours are contained in the unit rgb coordinate parallelepiped. For the ideal rgb monitor, the parallelepiped is simply the maximum volume inscribed parallelepiped in the object colour solid. The basis vectors of the parallelepiped define a spectral tripartition in parts that appear as r (appears red), g (appears green) and b (appears blue) (Bouma, 1948; Koenderink, 2010a; Schopenhauer, 1816; Schrödinger, 1920). Displays are such that the hardware implements this formal system. All rgb monitors closely approximate this,^2^ for technology naturally converges on the optimal solution. This explains the almost universally used rgb-based descriptions of screen colours (Foley, van Dam, Feiner, & Hughes, 2005).

The screen colours are an optimal representation of the object colours. For convenience, one adapts coordinates such that the unit coordinate parallelepiped becomes the unit rgb cube. Of course, any representation would do. Vision research prefers the CIE-Luv or CIE-L*ab colour spaces,^3^ whereas for end users (the great majority of users), the rgb system—in any of its variants—is the obvious choice. The rgb system is also especially elegant and convenient in formal colorimetric analysis. We use the rgb system in this article because the study is perhaps mainly of interest to end users.

The space of rgb screen colours has the topology of the interior of the 2-sphere $S^{2}$ (the parallelepiped volume), thus there is no unique serial order. This explains why putting coloured chips in a visually “natural” order can be a challenging task. It yields rather diverse results for different people in the case of small sets of colours, for instance, the well-known 11 colours that “will never be confused” (Boynton, 1989). Apparently, there is no such a natural order in that case. The colours are categorical. The reason is simply that the colorimetry implies no serial order to start with.

However, the screen colours distinct from the black–white axis have the topology of the interior of the 2-torus $T^{2}$ (the parallelepiped with a body diagonal removed). The angular variation encircling the black–white axis parameterises the “hues.”

If one constrains the colours apart from the black–white axis to those lacking any white or black content (Bouma, 1948; Koenderink, 2010a; Ostwald, 1919), they have the topology of the circle $S^{1}$ (a closed edge progression on the parallelepiped). The closed, polygonal arc of colours (excepting the black–white axis) then represents the equator of the rgb cube (a close approximation of the equator of the colour solid) in terms of the tripartite basis.^4^

Thus, the minimal set of screen colours for which a natural order exists is six, the colours y (yellow), g (green), c (cyan), b (blue), m (magenta) and r (red) in that periodic order (Koenderink, 2010a, 2018). These are the vertices of the aforementioned polygonal arc. With end users in visual art and graphical design, it is known as “the colour circle.” (The polygonal arc length [0–6] is often converted to angle [in radians 0–2π = 6.28 … ].)

In this article, we study the *discrimination along the colour circle*. The method we use is a simple sorting task.

The basic sequence ygcbmr can be refined by adding equal mixtures of adjacent pairs. For instance, the pair ry yields o (Orange) as the interpolant. This interpolant has maximum saturation in the rgb space. Thus, one obtains the triple roy. Repeating for all pairs yields a 12-step colour circle. In an analogous way, one obtains colour circles of various sizes, in the limit one obtains a continuous gradation.

Such colour circles are commonly used in colour pickers and applications aimed at artistic and visual design work. A continuous scale is commonly implemented, although a “limited palette” is universally recommended in art instruction (Quiller, 1989). If discretised, the graininess of the representation is typically decided upon arbitrarily or through user interface constraints.

It is of some practical interest to study the discrimination over colour circles of various sizes. The distribution of hues in these colour circles is fixed by the formalism mentioned earlier. It is reflected in all rgb-based systems.

**Figure 1.**
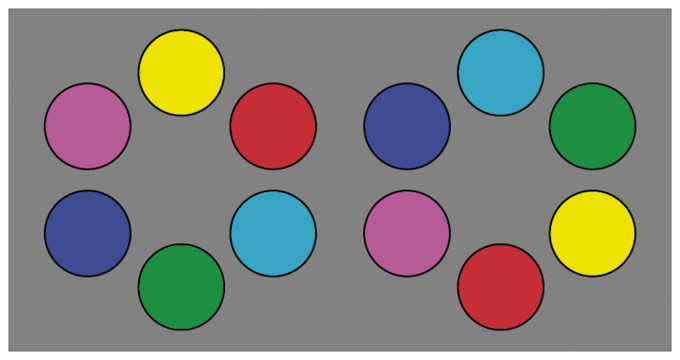
Which order is more “natural?” Most naive observers have no clue. You may well *know* the correct order, but do you *see* it? If not, then is the “correct” order an arbitrary convention? Indeed many uk-based people have root-learned “Richard Of York Gave Battle In Vain” (urban slang has more interesting mnemonics) to remember the order of spectral colours. (For most purposes you may safely forget the i [indigo] and v [violet].) All you need is to close the roygbiv sequence with p (purple) in order to get the hue circle.

Although there are formal reasons for the periodic sequence ygcbmr, whether this is reflected in phenomenology is an empirical matter. In practice, we find that many people are puzzled by the task of putting six chips {y, g, c, b, m, r} in a visually natural order (Figure 1). Apparently, the six “cardinal colours {y, g, c, b, m, r}” are phenomenologically only categorically different for the majority of people. However, many observers will agree with the order suggested by the rgb structure when it has been explained to them, understanding the colours as mutually *related*.

The sorting becomes much easier, to the point of looking “natural,” when more chips are interpolated. For a 12-point scale, most people readily arrange the sequence starting from a pile of chips.

**Figure 2.**
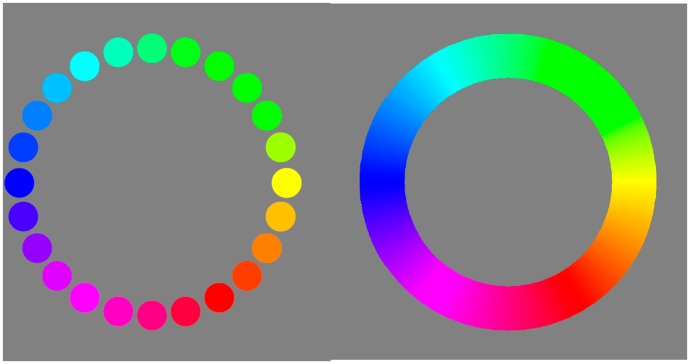
A 24-step colour circle compared with a continuous one. Are the chips in the discrete version all different? If so, how many hues are spotted in the continuous version? (The authors would venture “yes” to the first question and “six, perhaps seven” to the latter, which seems logically inconsistent, but fits the phenomenology.)

Interpolating even more colours indeed renders the task increasingly natural (Figure 2, left). However, as the number of interpolants is increased, one notices that most people agree on the order except for apparently random local disagreements. In such cases, similar chips are not so much seen as “related” as being “the same” or perhaps “the same for all practical purposes” (Davidson & Friede, 1953, p. 581/2) and are simply confused, at least at first blush. Apparently, one runs into a limit of resolution.

This is especially evident when a discrete scale is compared with a continuous scale (Figure 2, right). The chips of the 24-step scale appear distinct in hue, but how many hues does one spot in the continuous colour circle? In the latter case, one certainly cannot name all the colours (the reason why artificial systems were developed for naive users; Munsell, 1905; Munsell, 1912; Ostwald, 1919; Syme & Werner, 1814), but even in the 24-step scale, many people will find this difficult (Berlin & Kay, 1969; Miyahara, 2003); moreover, people are by no means the same in these respects (Kuehni, 2004). It implies that the chips are not really individuals, which might be considered to affect ordering qualitatively.

We are mainly interested in the sensitivity to colour order in natural contexts, where a natural context might be the display seen by a digital artist or designer (Quiller, 1989). In order to study this, we let participants order colour circles with various numbers of equi-spaced chips on an electronic display.

**Figure 3.**
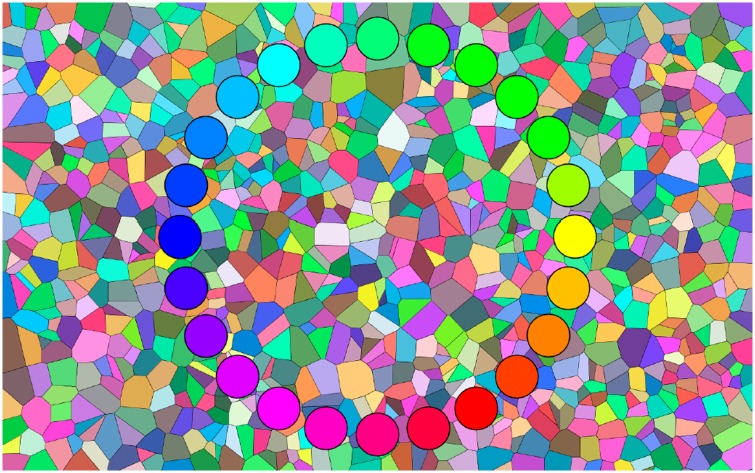
A 24-step colour circle in perfect sorting order. The background is a random pattern composed of all rgb colours. This background pattern is shifting all the time, changing very markedly at a temporal scale of seconds. We number the steps starting at yellow (1), moving in the direction of green. In this case, the indices run from 1 (yellow) to 24 (yellowish orange).

All chips are displayed at any given time. We only impose the constraint that the chips be geometrically ordered at equi-spaced locations on a circle. The background is a random pattern composed of all rgb colours (Figure 3). The statistics is explained in earlier studies (Koenderink, 2010b; Koenderink & van Doorn, 2017). This background is refreshed at a rate of 10 Hz, although somewhat gradually in order to prevent flicker. It is continually shifting and changing structure, effectively preventing comparison.

The densest subdivided colour circle in the experiment contains 60 chips. For such a size, participants already commit quite a few errors. Errors can be displacements by several steps, median displacement being two, upper quartile four.

With such an error rate, it becomes of much interest to study the distribution of errors over the colour circle. That is indeed the major objective of this study. Another aim is to arrive at an estimate of the desirable resolution for practical colour wheels used in the visual arts.

## Methods

### Equipment

The display was the LCD screen of an Apple MacBook Pro 15″ (mid 2007 model). It was linearised using Bergdesign Supercal (version 1.2.4). Photometric data on the display (as measured with a X–Rite ColourMunki photo-spectrometer) are (using conventional cie xyL coordinates; Wyszecki & Stiles, 1967):

**Red**  *x* = 0.5995, *y* = 0.3406, *L* = 68.9,

**Green** *x* = 0.3259, *y* = 0.5723, *L* = 197.4,

**Blue**    *x* = 0.1534, *y* = 0.1346,  *L* = 53.2.

These colorimetric data allow precise reproduction of our experiment. However, our results should reproduce (plus or minus some slop) on any modern display unit. Because of fundamental colorimetric reasons, all modern display units converge on the same red, green and blue components, the main difference will be in total radiant power and various technicalities that hardly matter for the present purposes.

The screen was binocularly viewed from a distance of about 57 cm and subtended about 32° × 20°. Experiments were done in a darkened room, thus the background pattern determines the adaptation level.

### Participants

A group of 15 participants was recruited at the University of Leuven, it consisted of PhD students, postdocs and technical or administrative staff. All volunteered, none had experience with experiments involving colour and some had no experience with formal experiments in vision science. They were tested for normal trichromacy using the conventional Ishihara test (Ishihara, 1917).

### Experiment

**Figure 4.**
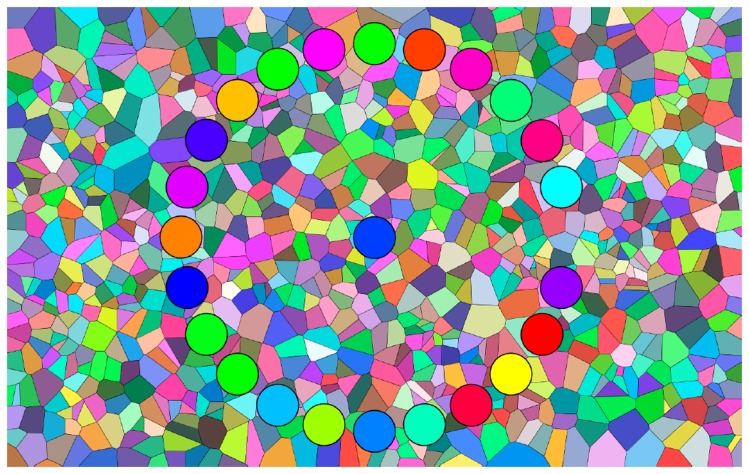
A stage in the sorting task. Here, the colour circle is still in major disarray (perfect sorting order shown in Figure 3).

A typical display is shown in Figure 4. It shows a randomised colour circle in the process of being ordered.

The participant can click on any chip that is seen to be out of order. The chip will magically move to the centre, leaving a gap (the situation illustrated in Figure 4). The participant may then move this chip (using the mouse) and drag it to its preferred location. The chips at either side of the preferred location will magically move aside to make place for the dragged item and the gap that was left by picking that chip in the first place vanishes. This process is iterated until the participant declares that the colour circle is in perfect sorting order.

Notice that the observers have to look back and forth between distinct locations all the time (Koenderink, van Doorn, & Ekroll, 2016), so memory colours no doubt play an important role in the process. Never can two chips be “directly compared” in a sense that has become a convention in regular psychophysics, the typical examples are the usual bipartite displays.

## Results

**Figure 5.**
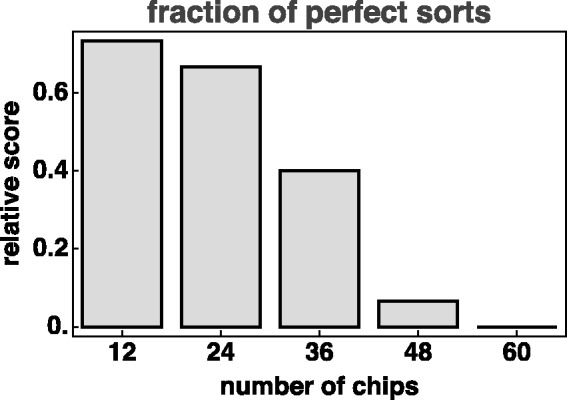
The fraction of observers that managed to do a perfect sort as a function of the number of chips on the hue circle.

We primarily focus on the 60-hue case, although we collected full data on 6-, 12-, 24-, 36-, 48- and 60-step colour circles. (The six-step case was used for initial practice, so we do not consider it further.) The coarser colour circles mainly serve as a good training for our participants. As stated earlier, a six-step scale tends to be problematic for naive observers, it appears like a mere bag of marbles to them. In contradistinction, quite a few people will sort a 24-step colour circle at the first try, without any glitches. No participant perfectly sorted the 60-step circle. The fractions of observers that managed to perform a perfect sort are shown in Figure 5 as a function of the total number of steps of the colour circle.

Indeed, colour circles of 24 steps are easy enough to sort for most of the observers, but there will likely occur a few confusions. Even more articulated colour circles are hard to display nicely on current display units due to lack of display space. We judge that the 60-step case is about right for the present purpose.

**Figure 6.**
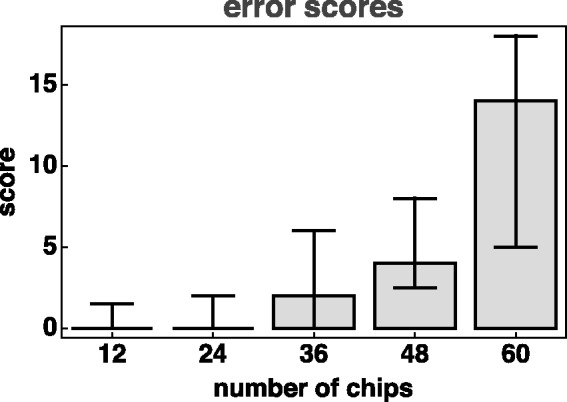
Error scores. We show medians and interquartile ranges. Notice that the score penalises both the number and the amplitude of the mismatch.

All participants are at least partly confused several times. For the 60-step hue circle, we find about 15% mismatches. Mismatches may be significantly larger than single-step confusions, we find mismatches up to five. We define an error score as the sum of the products of the number of mismatches of a given amplitude with that amplitude (Figure 6).

The mismatches turn out to be non-uniformly distributed, although in a roughly regular pattern.

### Analysis

**Figure 7.**
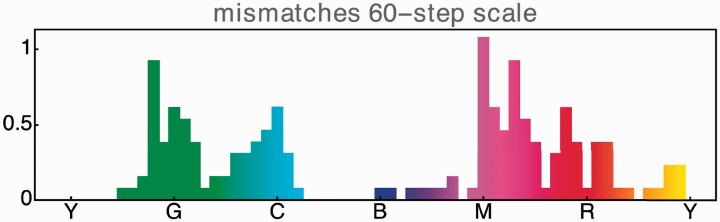
The average mismatches for the largest cluster. (Vertical scale represents the number of steps.)

It is a priori likely that there will be some outliers in a random group of naive observers. To clean the data, we performed a cluster analysis on the raw data, using an angular distance metric for the 60-dimensional response vectors, a spectral clustering method and a search for at most three clusters. We find cluster sizes of {13, 1, 1} and we keep the largest cluster.^5^ Constraining to the largest cluster renders the data a trifle less noisy. The mean results for the major cluster are presented in Figure 7.

By cursory inspection of Figure 7, one sees that the major variation occurs at a frequency corresponding to two cycles over the colour circle.^6^ This dominant variation peaks in the green-cyan and magenta-red. Least confusions are in the yellow and the blue. Thus, the confusions mainly occur on the boundary of the “cool” and the “warm” hues.

## Conclusions

Many human observers are able to sort a 24-step colour circle perfectly, although it takes some dedication. Finer grained hue circles give rise to sorting errors, even in very experienced observers. The patterns of such errors is quite uneven, the errors accumulate in the green-cyan and magenta-red regions. We discuss a few relations to conventional colour science and then move on to discuss consequences that might matter in praxis.

Notice that (quantified in the Methods section) the luminances are in the ratio *L_R_*:*L_G_*:*L_B_* = 21:62:17 (adding up to 100). The colour circle is in no way equiluminant, the luminance variation over the colour circle is as large as 4.9 (*L_Y_*/*L_B_*). Indeed, an equiluminant representation would be entirely useless from an applications perspective, for the “yellow” would have to be degraded to a dark brown. We expressly point this out, because a hue discrimination study (like sorting) without equiluminant constraint evidently goes squarely against the grain of acceptable methods in vision science.

The relevant literature mainly focusses on the use of the Farnsworth 100-hue test as a clinical tool (Craven, 1993; Farnsworth, 1943; Kinnear & Sahraie, 2002; Mäntyjärvi, 2001). This test has been designed to make it as easy as possible, by giving participants only short sequences to sort at any given time and by constraining chips to be viewed perfectly side-by-side and in a neutral context. Most people will make at least 10 errors in sorting the 100 chips.

The sensitivity to colour order is likely to be much lower in a more natural (more varied) context and with chips not necessarily perfectly adjacent. Indeed, our data reveal a rather lower discriminability than the Farnsworth 100-hue test suggests.

In a recent study (Koenderink, van Doorn, & Gegenfurtner, 2018), we used methods that might be said to have at least *some* affinity to the present method. It remains one of the few available sources of this type of data. Those data are squarely in the present ball park,^7^ given the differences of methods and quantification used. This is, of course, much as expected.

Another option is to convert the data to dominant wavelengths. This is not a particularly attractive format, but it has the virtue of mimicking historical data (starting with von Helmholtz, 1867). It is at least of academic interest to compare the sorting results to wavelength discrimination data.

Unfortunately, one notices quite a divergence in the available data. Numerous factors might play a role in that (Noorlander, Heuts, & Koenderink, 1980, 1981; Noorlander, Koenderink, Den Olden, & Edens, 1983; Thomson & Trezona, 1951; Zhaoping, Geisler, & May, 2011). For technical, methodological reasons, we prefer the Van Esch data of 1984 (Van Esch, Koldenhof, van Doorn, & Koenderink, 1984). We find an estimate that suggests a resolution that is perhaps similar to the estimate from the present sorting experiment, although the comparison is rather shaky.^8^

An in-depth investigation is difficult due to a variety of factors. Indeed, the attempt to relate the sorting results to wavelength discrimination data is perhaps abortive. One problem is that the wavelength discrimination data refer to equiluminance, whereas the colour circle has a luminance variation of about a factor of five.^9^

The conventionally quoted numbers of a million to 10 million discriminable colours (Wyszecki & Stiles, 1967) is mainly based on the data of MacAdam and Brown (Brown & MacAdam, 1949; MacAdam, 1942). Such numbers suggest resolution estimates that are one or two orders of magnitude better than our sorting results.^10^ Thus, such estimates are entirely non-realistic for mundane tasks like sorting chips. One reason might be that these numbers relate to a large (at least two decades of luminance) range of radiance levels. The present data by design apply to real-world tasks involving screen colours.

Yet another comparison would be with cie2000 distances between adjacent chips along the colour circle. This turns out to yield a qualitatively wrong result as the dominant variation has three cycles over the colour circle. Regions of large confusion would be near the red, green and blue. This is flatly contradicted by the results shown in Figure 7. The overall resolution seems comparable with our sorting results.^11^

From a pragmatic perspective, a hue selection interface using 24 steps is easy to use and offers ample resolution in most applications. It is about the resolution offered by deluxe sets of colour pencils. A 36-step interface is already far less easy to use, because adjacent samples appear very similar and are easily confused at first blush, whereas the increased resolution will rarely be an advantage in drawing or painting.

Both the Munsell and Ostwald colour circles were designed to find use in the applied arts (Munsell, 1905; Munsell, 1912; Ostwald, 1919). The typical Munsell hue scale has forty hues, not significantly different from 36, whereas Ostwald eventually settled on a 24-step colour circle (see especially Bouma, 1948). The Quiller colour circle, which is in common use by watercolour artists, is slightly more detailed. However, it is special because its “chips” stand for actual pigments, which makes a great difference to the painter (Quiller, 1989). Even when the hues are indiscriminable, the physicochemical properties of a pigment may indicate its use in particular settings.

A 36-step colour circle may well prove worthwhile in precise retouching tasks, but it is overkill for most artistic applications. Even higher resolutions will hardly ever make sense.

## Notes

1 For instance, “RGBColor[1,1,1]” (Mathematica) or “color(#FFF)” (Java) in familiar computer code.

2 This was definitely the case for the classical crt-based monitors. The most recent developments in display technology will slightly complicate this discussion, although the main conclusions still hold. Details are not relevant to the discussions in this article.

3 CIE-L*ab is actually a non-linear representation, thus does not allow immediate colorimetric calculations.

4 In either case, “equator” implies maximum “distance” from the black–white axis. In an affine space, typical for colorimetry, this implies the tangency with the narrowest circumscribed cylinder with generators parallel to the black–white direction.

5 In any study involving “naive subjects,” there is certain to be a fraction of at least 10% that is evidently “noise,” due to a variety of—mainly irrelevant—factors. We consider it as a good practice to restrict the analysis to the major cluster in such cases, although keeping all data should not change the final conclusions. This was also checked in this case. Keeping all data just for the sake of good manners goes against the grain of common sense science.

6 A Fourier analysis reveals that this component accounts for 40% of the spectral power. (In such a Fourier analysis one ignores the dc-component, which is irrelevant.) For such an analysis, one prefers the standard deviations of the apparent fuzziness of the locations of the chips, whereas the experiment yields probabilities of confusing the regular order. It is possible to estimate the standard deviation in the location from the observed probabilities. If the probability of confusing two adjacent chips is *P*, one finds that the standard deviation *σ* in the distribution of the apparent location of a chip is$\sigma (P)=\frac{1}{2{\text{erf}}^{-1}(1-2\text{P})}$.This allows a proper quantitative analysis of the topological disorder.

7 From Figure 10, left of Koenderink et al. (2018), we find that the ygcbmr locus may offer about 65 discriminable steps.

8 For the Van Esch data, we estimate about 26 discriminable steps over the spectrum by numerical integration of the just noticeable differences over the spectrum. The spectral range is the bcgyr part of the colour circle, it misses the bmr range, thus covers only two thirds. Hence, we obtain an (admittedly shaky) estimate of about 40 discriminable steps from the wavelength discrimination data. The wavelength discrimination data correlate better than 0.9 with the sorting data over the spectral range.

9 The luminance has a complicated variation over the colour circle. The dominant Fourier component has one cycle, and another major component has three cycles along the colour circle.

10 The numbers 10^6^ to 10^7^ suggest estimates of 600 to 1,300 discriminable steps along the colour circle.

11 Using the cie2000 distance metric, we find that the number of discriminable steps over the colour circle (the polygonal arc ygcbmry) is 2.814 … times the number of discriminable steps along the grey axis (the black-white body diagonal), which seems to be about right.
